# Toxoplasmosis in Pregnant Women and HIV/AIDS Patients in Ethiopia: A Systematic Review and Meta-Analysis

**DOI:** 10.1155/2019/4670397

**Published:** 2019-10-01

**Authors:** Daniel Getacher Feleke, Angesom Gebreweld, Gashaw Zewde

**Affiliations:** ^1^Department of Medical Laboratory Science, College of Medicine and Health Sciences, Wollo University, Dessie, Ethiopia; ^2^Department of Medical Laboratory Science, Ataye District Primary Hospital, Ataye, Ethiopia

## Abstract

**Background:**

Although *Toxoplasma gondii* infection in immune-competent individuals is usually asymptomatic or causes a mild flu-like illness, it may become severe and can occasionally be fatal in immune-compromised people, such as AIDS patients or pregnant women.

**Method:**

Electronic English databases (Pubmed, Google Scholar, Science Direct, and Scopus), parasitology congresses, and theses of Ethiopian medical universities, were systematically searched (published or unpublished data). Full-length articles and abstracts were collected using keywords such as *Toxoplasma gondii*, Toxoplasmosis, pregnant women, HIV/AIDS, and Ethiopia.

**Results:**

Analysis of seroprevalence estimates was pooled using a random effects meta-analysis. Seventeen studies were included in the present systematic review and meta-analysis. One of these studies reported seroprevalence of *T. gondii* in HIV/AIDS patients and pregnant women. In this review, a total of 4,030 individuals were included and analyzed. The pooled prevalence of *T. gondii* in this review was 81.00% (95% CI = 69.10–89.78). Sub-group analysis showed that 2,557 pregnant women were evaluated. In pregnant women, the pooled sero-prevalence was 71.2 (95% CI = [51.9%, 87.1%]. In HIV/AIDS patients, 1,473 individuals were evaluated and the pooled seroprevalence was 88.45 (95% CI = 80.87%–94.31%).

**Conclusion:**

This systematic review and meta-analysis identified a high seroprevalence of *Toxoplasma* infection of 81% among immunocompromised patients. Scaling up prevention and control methods mainly strengthening educational efforts are necessary to avoid reactivation and to stop the spread of *T. gondii* infection.

## 1. Introduction

*Toxoplasma gondii *is one of the most prevalent obligate intracellular protozoan parasites of man and livestock [1–5]. Feline species are definitive hosts for *Toxoplasma gondii* and a wide range of animals serve as intermediate hosts. Globally, it is estimated that about one-third of the population is infected with *T. gondii* [3]. Although *T. gondii* infection in immune-competent individuals is usually asymptomatic or causes a mild flu-like illness [4], it may become severe and can occasionally be fatal in immune-compromised people, such as AIDS patients or pregnant women [2, 6]. *Toxoplasma gondii* infection in AIDS patients and pregnant women causes severe encephalitis, and neurologic diseases, and can affect the heart, liver, inner ears, and eyes (chorioretinitis) [2]. Infection with *T. gondii* during pregnancy can result in spontaneous abortion, still-birth, fetal death, neonatal death, or various congenital defects, such as hydrocephalus, central nervous system abnormalities, and chorioretinitis [7]. HIV infected individuals are at risk of acute toxoplasmosis due to reactivation of *T. gondii*. It also causes cervical lymphadenopathy or ocular disease in HIV infected immune competent individuals.

Humans get infections with *T. gondii* after ingesting raw or undercooked meat, by ingesting cat-shed oocysts via contaminated soil, food or water; or trans-placental transmission [3–5, 7].

Serological methods used for *T. gondii* diagnosis such as enzyme-linked immunesorbent assay (ELISA) and indirect immunofluorescence assay (IFA) are considered as gold standard for the detection of *Toxoplasma*-specific antibodies (IgG or IgM) [4].

In Ethiopia, human toxoplasmosis infection is a neglected disease [7]. The prevalence of *T. gondii* infection in Ethiopia has been reported in some populations; mainly in HIV infected individuals, pregnant women, child bearing age women, and in general population [4].

For appropriate diagnosis, treatment, and control of *T. gondii* infections, information about the seroprevalence of toxoplasmosis in different special populations is very important. So, this systematic review of the literature aimed to evaluate the sero-prevalence of *T. gondii* among pregnant women and HIV/AIDS patients in Ethiopia.

## 2. Methods

### 2.1. Literature Search and Data Extraction

Relevant literatures were searched on Pubmed, Pubmed central, Google scholar, science direct databases, Scopus, theses of Ethiopian medical universities, and Ethiopian journals such as Ethiopia journal of health development and proceedings of professional associations from May, 2018 to June, 2018. Full-length articles and abstracts were collected using keywords such as *Toxoplasma gondii*, Toxoplasmosis, pregnant women, HIV/AIDS, and Ethiopia. These key words were also used in combination using boolean operators. Overlapped articles found in more than one databases were excluded. Annual research conferences and preceding of Ethiopian medical laboratory association (EMLA), and Ethiopian public health laboratory associations' (EPHLAs) records were reviewed to identify relevant unpublished articles.

The reference lists of published studies included in this meta-analysis were searched for additional articles. Two authors independently reviewed all collected studies for eligibility and they extracted the required information based on the objective this meta-analysis.

### 2.2. Data Collection

**Figure 1:**
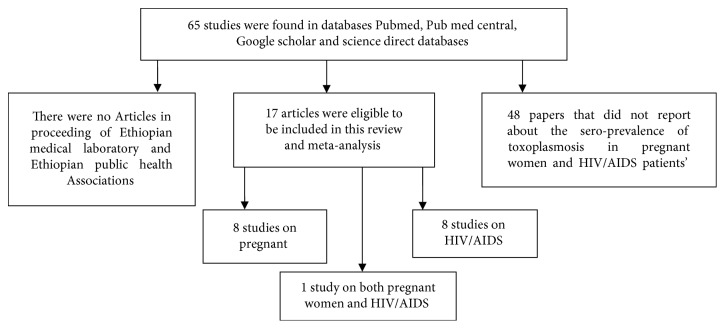
Briefly shows the search process in this review article.

Pre-designed data extraction form was developed by authors based on the objective of this review. Studies conducted in Ethiopia to estimate the sero-prevalence of *T. gondii* infection in pregnant women and HIV/AIDS patients were included. All collected studies were assessed by two authors for eligibility to be included in this review. In case of disagreement on the eligibility of identified studies, authors held discussion to resolve the issue by considering the quality of identified study and the aim of the present review. Studies performed in study groups other than pregnant women, and HIV/AIDS and those studies that did not use random sampling methods as a sampling technique were excluded (Figure 1). Information about year of publication, first author, study area, study design, total sample size, number of sero-positive individuals, and laboratory methods used for *T. gondii* diagnoses was carefully investigated.

### 2.3. Statistical Analysis

Point estimates and 95% confidence intervals of seroprevalence of all included studies were calculated. An overall seroprevalence and group-specific sero-prevalences were calculated among HIV/AIDS patients and pregnant women. Heterogeneity among studies was visualized using a forest plot chart. The *I*^2^ and Cochran's Q tests were used to quantify the variations between studies. The heterogeneity was considered not significant when *P* > 0.1 and *I*^2^ < 50%. Due to the heterogeneity of studies included in this meta-analysis, random effect model was used by considering the included studies used random samples from a population. This meta-analysis was performed using the trial version of StatsDirect statistical software (https://statsdirect.com).

## 3. Results

Out of 65 studies from literature searches using key words, 17 studies were eligible and included in the present systematic review and meta-analysis. Eight studies described the sero-prevalence of *T. gondii* in pregnant women, whereas 8 studies reported *T. gondii* infection in HIV/AIDS patients. There was also one study which reported seroprevalence of *T. gondii* in HIV/AIDS patients and pregnant women. This study was considered in both pregnant women and HIV/AIDS patient groups during analysis. The study designs of all eligible and included studies were cross-sectional. In this review, Enzyme-linked immunosorbent assay (ELISA) and latex agglutination slide tests were the commonly used methods for the detection of *T. gondii* antibodies. The higher sero-prevalence of *T. gondii* (96.7% and 93.6%) was reported in HIV/AIDS patients from Mizan Aman Hospital and selected areas of Ethiopia, respectively. On the other hand the lower seroprevalence (18.5% and 34.9%) reported in this study was in pregnant women from Felege Hiwot Hospital from Jigjiga Hospital (Tables 1, 2 and 3).

Detection of *T. gondii* IgG and IgM is one of the commonest serologic diagnoses. IgG detection indicates either past or present infection while the detection of IgM showed early infection of *T. gondii*. Based on IgG assessment, most of the current reviewed studies reported above 95% *T. gondii* IgG sero-prevalence. In general, the sero-prevalence of IgG is higher than IgM in pregnant women and HIV/AIDS patients (Tables 1 and 2).

**Figure 2:**
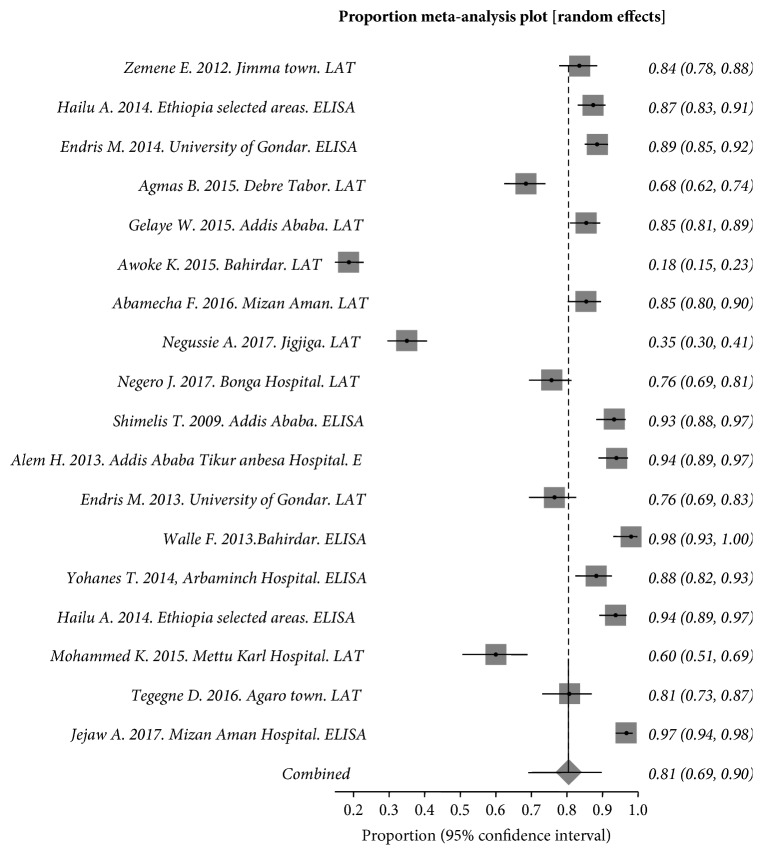
Forest plot diagram of studies showing seropositivity to *T. gondii* in pregnant women and HIV/AIDS patients from Ethiopia.

In general, in this review a total of 4,030 individuals were included and analyzed. The pooled prevalence of toxoplasmosis in this review was 81.00% (95% CI = 69.10–89.78). The *Q* statistic and I_2_ (inconsistency) were Cochran Q = 1,233.145293 (df = 17, *P* < 0.0001) and I_2_ (inconsistency) = 98.6% (95% CI = 98.5%–98.8%) (Figure 2). In this meta-analysis and systematic review, time interval sub-group analysis was performed to observe *T. gondii* sero-prevalence change from old studies to recent studies. Included studies from 2009 to 2017 were grouped in five years' interval. The pooled sero-prevalence of *T. gondii* from 2009 to 2013 was 90% (95% CI = 82%–96%) while it was 76% (95% CI = 61%–89%) in recent studies (2014–2017). Majority of the studies were conducted in recent years (2014–2017). Sub group analysis showed the sero-prevalence of *T. gondi* is decreased in recent years.

**Figure 3:**
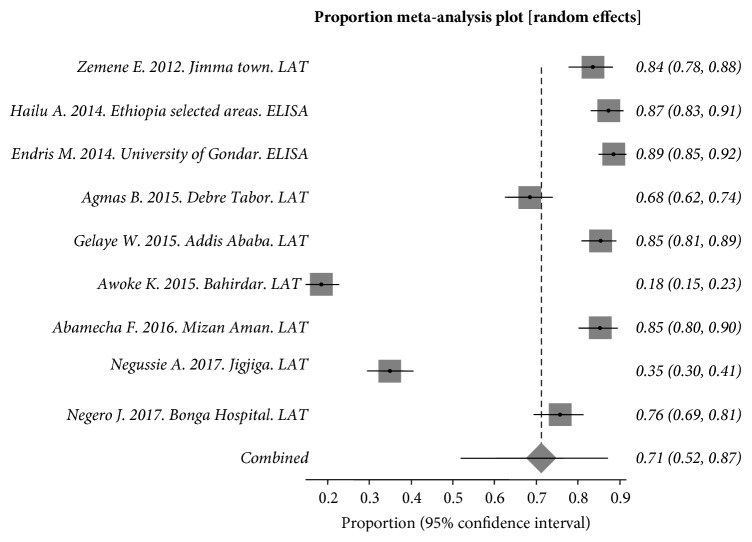
Forest plot diagram of studies showing seropositivity of *T. gondii *in pregnant women from Ethiopia.

Sub-group analysis showed that 2,557 pregnant women were included and evaluated. Random effect analysis was used due to the heterogeneity between studies. The *Q* statistic and the pooled sero-prevalence were Cochran Q = 845.808548 (df = 8, *P* < 0.0001) and 71.2 (95% CI = [51.9%, 87.1%] respectively (Figure 3). I_2_ (inconsistency) = 99.1% (95% CI = [98.9%, 99.2%] (Figure 3).

**Figure 4:**
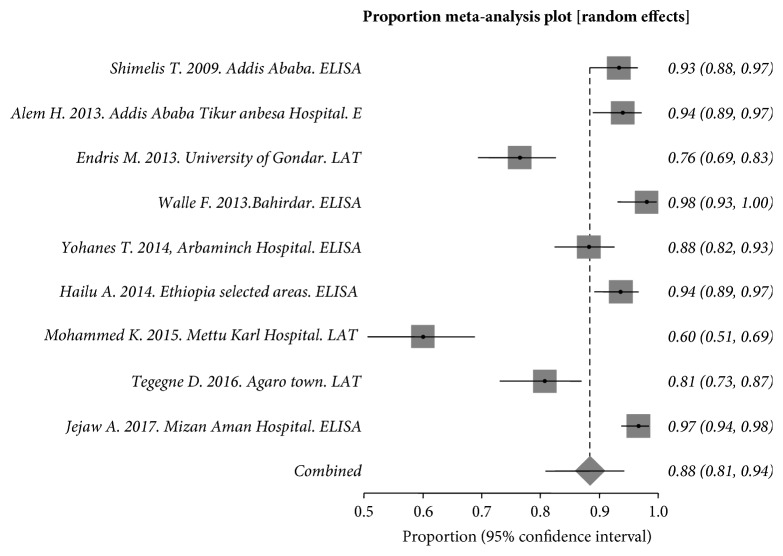
Forest plot diagram of studies showing seropositivity of *T. gondii *in HIV/AIDS patients from Ethiopia.

In HIV/AIDS *patients*, 1,473 individuals were included and evaluated. The *Q* statistic and the pooled seroprevalence were Cochran Q = 136.254836 (df = 8, *P* < 0.0001) and 88.45 (95% CI = 80.87%–94.31%) respectively. I_2_ (inconsistency) = 94.1% (95% CI = 91.5%–95.7%) (Figure 4).

Begg and Egger tests were used for evaluating publication bias. Significant publication bias was not revealed in studies included about pregnant women (*P*-value >0.05). There was significant bias among studies included about HIV/AIDS patients (*P*-value >0.05).

## 4. Discussion

*Toxoplasma gondii* infection is usually asymptomatic in immune-competent individuals or causes a mild illness [4]. However, it may become severe and can occasionally be fatal in immune-compromised people, such as HIV/AIDS patients or pregnant women [2, 6].

This is the first systematic review and meta-analysis of toxoplasmosis in pregnant women and HIV/AIDS patients in Ethiopia. Among 2,557 pregnant women in Ethiopia, the sero-prevalence of *T. gondii* was 71.1%. Furthermore, the seroprevalence of *T. gondii* in 1,473 HIV/AIDS patients was 88.45%. Immune compromised patients are at high risk of toxoplasmosis reactivation [17]. The finding of this meta-analysis showed that the seroprevalence of *T. gondii* in pregnant women and HIV/AIDS patients was higher than 70%, and there is a risk of reactivation of *T.gondii* in this group of population. Toxoplasmosis is an opportunistic disease that can cause very severe illness and fatality in immune compromised individuals. Pregnant women and HIV/AIDS patients are immune deficient individuals. This immune inefficiency could be the main risk factor for the higher sero-prevalence of *T. gondii*.

Toxoplasmosis is mainly transmitted to human by ingestion of oocysts with contaminated food and drinks, by consumption raw or undercooked meat contain bradyzoites. It can also be transmitted during blood transfusion and organ transplantation.

Immunocompromised patients are highly vulnerable groups for toxoplasmosis infection due to debilitated immune system.

In Ethiopia, human toxoplasmosis infection is a neglected disease [7]. Some studies were conducted on the seroprevalence of toxoplasmosis. The study group of majority of the studies was on pregnant women, general population, and HIV/AIDS patients. Toxoplasmosis in this group of the population can have devastating consequences. For instance, congenitally transmitted toxoplasmosis in pregnant women can have serious disease in fetus including chorioretinitis and neurological complications. In HIV/AIDS patients beside the virus toxoplasmosis may play a role in immune suppression and fasten the onset of AIDS symptoms.

Serological tests are considered as the gold standard in the diagnosis of *T. gondii* infection. Due to some shortcomings in serologic tests and the variation of serologic methods used, the seroprevalence of *T. gondii* infection might be varied.

In Ethiopia, *T. gondii* seroprevalence studies in pregnant women and HIV/AIDS mainly used latex agglutination test and Enzyme linked immune sorbent assay (ELISA) methods for diagnosis. The highest prevalence of toxoplasmosis was observed in individuals diagnosed with ELISA. For instance, in HIV/AIDS patients, 96.70% sero-prevalence of *T. gondii* was observed using ELISA. The incidence and prevalence of *T. gondii* infection varies depending on geographic areas, weather conditions, and age groups in Ethiopia.****In Ethiopia, a review studied on the sero-prevalence of *T. gondii* in general population revealed the overall pooled prevalence was 74.73% [18]. The seropositivity in pregnant women and HIV/AIDS patients in the present study was 71.1% and 88.45%, respectively. The seroprevalence in HIV/AIDS patients was higher than reported from general population and pregnant women sero-prevelence in the present study.

## 5. Conclusions

As far as we know and our literature search, this is the first systematic review and meta-analysis that provides information about the seroprevalence of *T. gondii* in pregnant women and HIV/AIDS patients in Ethiopia. Our finding indicates that researchers must give attention to the seroprevalence of toxoplasmosis in immunocompromised patients especially in HIV/AIDS patients, and pregnant women. Scaling up prevention and control methods and mainly strengthening educational efforts are necessary to avoid reactivation and to stop the spread of *T. gondii* infection.

## Figures and Tables

**Table 1:** The baseline characteristics of the studies included in this literature search.

Author	Publication Year	Study area	Study group	No of cases	Total sample size	Sero-prevalence	Age range	Serologic test	Ref
Shimelis T.	2009	Addis Ababa	HIV/AIDS patients	154	165	93.3%	20–66	ELISA	[8]
Zemene E.	2012	Jimma town	Pregnant women	168	201	83.6%	17–35	ELISA	[3]
Walle F.	2013	Bahir Dar	HIV/AIDS patients	101	103	87.4%	NA	ELISA	[9]
Alem H.	2013	Addis Ababa (Tikur Anbesa Hospital)	HIV/AIDS patients	141	150	94.0%	NA	ELISA	[10]
Endris M.	2013	University of Gondar Hospital	HIV/AIDS patients	130	170	76.5%	NA	LAT	[6]
Hailu A.	2014	Ethiopia selected areas	HIV/AIDS patients	178	190	93.6%	NA	ELISA	[11]
Yohanes T.	2014	Arba Minch Hospital	HIV/AIDS patients	150	170	88.2%	NA	ELISA	[12]
Hailu A.	2014	Ethiopia selected areas	Pregnant women	256	293	87.3%	NA	ELISA	[11]
Endris M.	2014	University of Gondar Hospital	Pregnant women	341	385	88.6%	NA	LAT	[6]
Gelaye W.	2015	Addis Ababa	Pregnant women	246	288	85.4%	18–42	LAT	[4]
Awoke K.	2015	Felegehiwot Hospital, Bahir dar	Pregnant women	71	384	18.5%	NA	LAT	[5]
Agmas B.	2015	Debre Tabor Hospital	Pregnant women	180	263	68.4%	18–44	LAT	[1]
Mohammed K.	2015	Mettu Karl Hospital	HIV/AIDS patients	72	120	60.0%	NA	LAT	[13]
Abamecha F.	2016	Mizan Aman Hospital	Pregnant women	198	232	85.3%	NA	ELISA	[7]
Tegegne D.	2016	Agaro town	HIV/AIDS patients	109	135	80.7%	NA	LAT	[14]
Negero J.	2017	Bonga Hospital	Pregnant women	159	210	75.7%	15–44	LAT	[15]
Negussie A.	2017	Jigjiga Hospital	Pregnant women	105	301	34.9%	NA	LAT	[2]
Jejaw A.	2017	Mizan Aman Hospital	HIV/AIDS patients	261	270	96.7%	18–49	ELISA	[16]

NA: not applicable.

**Table 2:** Baseline characteristics of included studies (based on IgG assessment).

Author	Publication year	Study area	Study group	No of cases	sample size	IgG cases	Sero-prevalence	Age range	Serologic test	Ref
Zemene E.	2012	Jimma town	Pregnant women	168	201	163	97.0%	17–35	ELISA	[3]
Walle F.	2013	Bahir Dar	HIV/AIDS patients	101	103	90	89.1%	NA	ELISA	[9]
Alem H.	2013	Addis Ababa Tikur Anbesa Hospital	HIV/AIDS patients	141	150	141	100.0%	NA	ELISA	[10]
Hailu A.	2014	Ethiopia selected areas	Pregnant women	256	293	247	96.5%	NA	ELISA	[11]
Endris M.	2014	University of Gondar Hospital	Pregnant women	341	385	341	100.0%	NA	LAT	[6]
Hailu A.	2014	Ethiopia selected areas	HIV/AIDS patients	178	190	172	96.6%	NA	ELISA	[11]
Abamecha F.	2016	Mizan Aman Hospital	Pregnant women	198	232	191	96.5%	NA	ELISA	[7]
Jejaw A.	2017	Mizan Aman Hospital	HIV/AIDS patients	261	270	255	97.7%	18–49	ELISA	[16]

NA: not applicable.

**Table 3:** Baseline characteristics of included studies (based on IgM assessment).

Author	Publication year	Study area	Study group	No of cases	Sample size	IgM positive	Sero-prevalence	Age range	Serologic test	Ref
Zemene E.	2012	Jimma town	Pregnant women	168	201	2	1.2	17–35	ELISA	[3]
Walle F.	2013	Bahir dar	HIV/AIDS patients	101	103	11	10.89	NA	ELISA	[9]
Hailu A.	2014	Ethiopia selected areas	Pregnant women	256	293	9	3.52	NA	ELISA	[11]
Hailu A.	2014	Ethiopia selected areas	HIV/AIDS patients	178	190	6	3.37	NA	ELISA	[11]
Abamecha F.	2016	Mizan Aman Hospital	Pregnant women	198	232	7	3.54	NA	ELISA	[7]
Jejaw A.	2017	Mizan Aman Hospital	HIV/AIDS patients	261	270	6	2.3	18–49	ELISA	[16]

NA: not applicable.

## Data Availability

The authors confirm that all data underlying the findings are fully available without restriction. All relevant data are within the manuscript.

## Abbreviations

HIV: Human immunodeficiency virus
AIDS: Acquired immunodeficent syndrome
ELISA: Enzyme linked immunosorbent assay
LAT: Latex agglutination test.
